# Gloucester Infirmary
*Midland Reporter, No. III.


**Published:** 1829-04-01

**Authors:** 


### XXXVII.

### GLOUCESTER INFIRMARY.*

### I. DISLOCATION OF THE FEMUR INTO THE ISCHIATIC NOTCH.

[Reported by W. FLETCHER.]

A stout man, ætatis 30, whilst ascending the stage of a show, fell to the ground on his left hip. He was brought to the hospital, and found by the house-surgeon with the foot turned in, the knee slightly bent, and the limb about an inch shorter than its fellow. The house-surgeon pronounced the accident, dislocation of the head of the femur into the ischiatic notch, and, four hours afterwards, when the surgeon, Mr. Fletcher, arrived, examination discovered the following symptoms. The whole limb was slightly rotated inwards—the knee somewhat bent and advanced—the trochanter major behind its natural place—the heel about an inch from the ground—the toe just touching it. Under these circumstances, Mr. Fletcher confirmed the house-surgeons's diagnosis, and ordered the patient to be bled to ℥xxx, and nauseated, by means of tartarized antimony in water. The patient, at the same time, was laid on his right side upon a low bedstead, covered with blankets, and placed between two posts, to which staples were affixed. The pelvis was well fixed by a band between the thighs and the upper staple, another band was carried round the pelvis and under the bedstead, and a slow extension was made by the pulley across the middle of the thigh. Having taken three grains of the antimony without the supervention of nausea, a tobacco enema was administered, and produced the desired effect. A jack-towel was now passed under the upper part of the affected thigh, and around the neck of an assistant, who attempted in this way to lift the head of the bone over the projecting brim of the acetabulum. Half an hour had been consumed in these attempts, and although no actual snap had been heard, the joint had assumed a more natural appearance, and the bandages, &c. were removed to examine its condition. It was evident that reduction had not been effected, and the patient vomiting freely, the apparatus was quickly re-adjusted, and extension a second time attempted. The rollers, however, got loose, and required to be once more removed, whilst the patient continued vomiting and extremely low. When the house-surgeon was gently rotating the limb, in order to re-apply the circular strap above the knee, a loud snap was heard, and the head of the bone was found to have re-entered the acetabulum, under this gentle manipulation, after it had resisted the former powerful and long-continued efforts.

"My father ascribed this unexpected event taking place without any extension, in this difficult case, to the utter loss of strength of the patient—the powerless condition of the muscles; indeed, the man was frightfully exhausted. My father further remarked, that facts are perpetually occurring, which put aside all rules. He had seen four cases of Luxation of the Femur backwards, reckoned the most difficult of all luxations of that bone to reduce, all reduced; one on the Pubis, though every care was taken, was not reduced; another into the Foramen Ovale, not reduced, though here no attempt had been made; and nine upon the Dorsum Ilii, one of which was not reduced. This catalogue of such cases, and their proportions to each other, is a confirmation of what Sir A. Cooper has stated to be, as near as can be, their relative proportions. In twenty cases, he says there will probably be twelve on the Dorsum Ilii; five in the Ischiatic Notch; one on the Pubis, and two in the Foramen Ovale. Mr. F. also remarked 'that though it is so desirable to weaken the system, and thence the resisting power of the muscles in difficult cases of Luxated Femur, yet nothing requires more experience and care than the administration of such powerful agents as Emetic Tartar and Tobacco, which, together with the violence of the operation, is sometimes too much for the powers of life to sustain.'"

In the latter observation we fully agree with Mr. Fletcher, indeed we should hesitate, in any case of dislocation, before we had recourse to so dangerous a drug

---

* Midland Reporter, No. III.

as tobacco. The emetic tartar, if given with patience, and combined with bleeding, produces depression and nausea enough, and occasionally, we suspect, even more than enough. In a case of dislocation of the humerus, bleeding to twenty ounces, and an ounce of the liquor antimonii tartarizati produced the most deadly faintness we ever remember to have witnessed. This patient subsequently died of phlebitis; but, whether the excessive nausea had any thing to do with the supervening inflammation of the vein that was opened, we cannot pretend to affirm.

### II. Large Lacerated Wound of the Perineum, Scrotum, and Urethra.

[Reported by J. R. Bedwell.]

A man, twenty-seven years of age, was brought to the Infirmary, October 8, 1827, in consequence of part of a house having fallen on him. A lacerated and gaping wound extended from the tuber of the ischium by the anus, along the perineum, and through part of the scrotum, exposing the bulb of the urethra. Considerable bleeding had occurred, but only an oozing took place on his admission. The lips of the wound were brought together by five or six sutures, a compress of lint and wet rags applied, and over them all a T bandage. Thirty drops of laudanum were also given in an ounce of mint water, and the patient ordered to be kept low. Next day no unfavourable symptoms had appeared, but the urine passed freely from the wound about the bulb of the urethra, in which there was evidently a large laceration. He took some castor oil that day, and was going on well on the 10th, when an attempt was made to introduce a flexible catheter into the bladder, but it could not be passed beyond the wound. Slight sloughs formed over the exposed part of the urethra, and were detached without any severe constitutional symptoms. On the 20th the patient made some water by the natural passage, and Nov. 10th, the external wound was healing fast, and the whole of the urine was voided the right way. A stricture formed about the rent in the urethra, which required the use of the bougie, but at the time of the report, the patient was nearly well, and about to be discharged from the Infirmary.

"Mr. Fletcher remarked 'that this was a case of torn up Perinæum and Urethra, cured by the simplest means. The dresser had carefully stitched up the wound, probably without knowing of the laceration of the Urethra. Nature, with her adhesive process, did all, as she does in Lithotomy, where the Urethra is sometimes greatly injured. Art was here required only to enlarge the point of the urethra, which was contracted from the injury. Gentlemen should understand that these cases of ruptured Urethra, from external violence, are very different from those which happen from disease.'"

This is certainly a very different case from that in which the urethra bursts behind the stricture, and occasions extravasation of urine. Suppose, however, that the urethra is broken by external violence, the skin and integuments being *sound*. The difference, then, would not be so great, indeed the cases would be similar, as far as the immediate effects were concerned. In both, the urine would get loose from its natural channel —in both, it would be infiltrated into the surrounding cellular membrane—in both, suppuration and sloughing of that membrane would occur, unless the surgeon made free scarifications. It is not the mere *mode* in which the urethra is opened that constitutes the important item in the case, but the circumstance of the integuments being broken or unbroken. If *they* are wounded the urine escapes, and is not injected into the cellular tissue, because the violence which ruptured the urethra, also *scarified* the external parts.

### III. Difficult Case of Lithotomy.

[Reported by R. Fletcher.]

Every one at present has his difficult case of lithotomy, though, fortunately, every one has not the curse of a Lambert to report it for him. The subject of the present case was a man, ætat. 50, admitted with the usual symptoms of stone, accompanied with such excessive irritability of the bladder, that Mr. Fletcher did not think it right to encourage him with very much hope from the operation, which, however, was performed at his own request. After cutting into the bladder, two large stones were discovered, which required the incision in the viscus to be enlarged, and required nearly half an hour to accomplish their extraction, although they had previously been broken. During the operation, the transversalis perinei bled nearly a quart, and the patient, in consequence, became so low and cold, that brandy was required to support him. Early next morning, he was seized with vomiting of high-coloured green matter, and rejected every thing administered in the way of medicine. Enemata were given without effect; the pulse was 120, and feeble—the tongue furred and white. In the evening he was so depressed as to feel convinced that he must die, and suffered, at the same time, from pain and tenderness in the hypogastrium. Leeches were ordered, but not applied till next morning, when a croton-oil pill was given, and thrown up. The pulse was now "uncommonly quick and feeble," and the tongue brown. In the afternoon the pulse was imperceptible, and brandy was given, in small and frequent doses, with the happiest effect. The pulse rose—he had stools in the course of the night, and has since been doing well. Mr. Fletcher considers that the brandy saved him, by maintaining the powers of life till the bowels acted. That the brandy saved the patient we have no doubt whatever; but whether the action of the bowels was of that importance Mr. F. would seem, by his remark, to imply, we are not so sure. From cases we have seen, we are led to suspect, that after excessive sanguineous evacuations or hæmorrhages, the bowels will be torpid, merely through the depression of the vis vitæ, consequent on the loss of blood. However that may be, Mr. Fletcher deserves great credit for the able manner in which he conducted so desperate a case to a fortunate conclusion.

---

